# Application and Development of QKD-Based Quantum Secure Communication

**DOI:** 10.3390/e25040627

**Published:** 2023-04-06

**Authors:** Junsen Lai, Fei Yao, Jing Wang, Meng Zhang, Fang Li, Wenyu Zhao, Haiyi Zhang

**Affiliations:** China Academy of Information and Communication Technology (CAICT), Beijing 100191, China

**Keywords:** quantum key distribution (QKD), quantum secure communication (QSC), application, standardization, verification

## Abstract

Quantum key distribution (QKD) protocols have unique advantages of enabling symmetric key sharing with information-theoretic security (ITS) between remote locations, which ensure the long-term security even in the era of quantum computation. QKD-based quantum secure communication (QSC) enhancing the security of key generation and update rate of keys, which could be integrated with a variety of cryptographic applications and communication protocols, has become one of the important solutions to improve information security. In recent years, the research on QKD has been active and productive, the performance of novel protocol systems has been improved significantly, and the feasibility of satellite-based QKD has been experimentally verified. QKD network construction, application exploration, and standardization have been carried out in China as well as other countries and regions around the world. Although QKD-based QSC applications and industrialization are still in the initial stage, the research and exploration momentum is positive and more achievements could be expected in the future.

## 1. Introduction

With the development of ability to produce, manipulate, and measure quantum states at the sub-atomic scale, the exploration of disruptive and innovative applications for the acquisition, processing, and transmission of information has been sped up [1]. Quantum information technology can break through the capability limits of classical information technology, and bring a quantum leap in computation power, network information security, measurement accuracy, etc. [2] Nowadays, quantum information science and technology, including quantum computing, quantum communication, and quantum sensing, has become one of the global hotspots for the high-tech industry [3,4].

Quantum communication realizing quantum state transmission or key distribution by using quantum superposition or entanglement effects, with the assistance of classical communication, can guarantee information theory security (ITS) at protocol level [5]. Quantum communication includes a variety of protocols and applications, such as quantum teleportation [6], quantum key distribution (QKD) [7], quantum secret sharing [8], quantum dense coding [9], and quantum secure direct communication [10], etc. Based on quantum teleportation and quantum memory and relay, the quantum information network, also known as the quantum Internet [11], can be built, which has become a focus for scientific research, but is still far from practical deployment and application. Quantum secure communication (QSC) using QKD to provide pre-shared keys for various cryptographic applications in the ICT system and network, typically combined with symmetric encryption, has entered industrial practice in the last decades [12].

Addressing the potential information security threats brought by quantum computing is the major interest for the industrial adoption of QKD-based QSC. Quantum computers capable of effectively running the Shor algorithm, which is predicted to appear around 2033 [13], can crack key establishment mechanisms of current public-key cryptography, ex RSA and ECDH algorithms. Standardization and migration efforts of post-quantum cryptography (PQC), expected to resist known threats of quantum computing, have continued to advance recently [14]. Compared with PQC, QKD protocols still offer unique and significant advantages of creating symmetric keys between remote locations, with information-theoretic security (ITS) guaranteed by quantum mechanics [15]. QKD-based QSC can ensure the security of symmetric key establishment, which will not be degraded over time, and also can achieve higher frequency of key updates, thus improving the overall security of encryption applications.

As one of the most influential and practical quantum information technologies, QKD-based QSC scientific research, application exploration, and industrial development have kept active and steady progress in the past decade. Several national quantum science and technology strategies have identified the QKD network as the initial stage in realizing the future quantum Internet [16], leveraging its advantages for network information security assurance [17,18]. A variety of novel QKD protocols and implementations continued to be optimized, and system performance such as maximum transmission distance and secure key rate had notable breakthroughs [19,20]. The integrated deployment and flexible networking of QKD and ICT systems and networks were explored [21]. Different types of QKD systems and encryption solutions have been commercialized by multiple vendors and service providers [22]. QKD network construction and demonstration application projects, mainly supported by public R&D funds, have been carried out in a number of countries and regions around the world [23]. Continuous innovation, application exploration, and commercialization practice indicate the importance of QKD-based QSC technology and application in the upcoming quantum era, which have been widely recognized and valued by government, academia, and industry.

In this paper, Section 2 briefly overviews the advances and trends in QKD technology research, Section 3 reviews the applications of QKD-based QSC in China, Section 4 summarizes the standardization of QKD and QSC conducted by the China Communications Standards Association (CCSA), Section 5 discusses the test and verification practices of QKD and QSC products and networks, and Section 6 summarizes application challenges for QKD-based QSC and the outlook for future development.

## 2. QKD Technology Research Advances

QKD is the most essential building block in QSC systems and networks that provides symmetric key sharing functions. QKD system performance, reliability and practical security are the decisive factors that directly affect whether QSC can be large-scale deployed and applied in practice [24]. The continuous progress and breakthrough in QKD technology research are the fundamental driving forces for the commercial application, exploration, and industrial development of QSC.

### 2.1. System Performance Enhancement

QKD systems can be realized based on various protocols and implementations with different strengths and limitations. Entanglement protocol-based QKD [25], which requires the generation and transmission of entangled photon pairs, has relatively limited system performance due to low entanglement yield and fidelity at the current state of the art, though it has better compatibility with the future quantum Internet architecture.

Preparation-measurement protocols-based QKD are more interested in research and application areas, which can be implemented using discrete variables (DV) or continuous variables (CV), and quantum state encoding can utilize different degrees of freedom of optical signal, ex polarization [26], phase [27], position, and phase difference between adjacent pulses [28], etc. The key rate of a typical point-to-point preparation-measurement QKD system is correlated to transmission efficiency. Due to the inherent loss limitation of optical fiber channels, it is difficult to exceed the single-span 500 km fiber transmission limit [29]. In addition, the imperfection of receiver-side detectors of preparation-measurement QKD systems may lead to side-channel security vulnerabilities and become a risk point for practical system security.

Since 2018, a twin-field (TF) QKD protocol has been proposed and received much attention [30], in which dual-end preparation and center measurement architecture can eliminate all side channel vulnerabilities at the detector, as well as increasing the theoretical secure key rate correlated to the square root of transmission efficiency and breaking the PLOB boundary of quantum channel capacity [31]. With the improvement of theories and protocols such as the sending-or-not-sending (SNS) protocol [32], the two-way classical communication (TWCC) method [33], and the active odd-parity paring method [34], TF-QKD has become one of the widely recognized next-generation long-range, high-security QKD solutions. A non-exhaustive list of recent typical QKD hero experiments is shown in Table 1.

In the latest record-setting experiment, a USTC team used an optimized four-phase twin-field protocol, combined with independent source phase-locking, channel phase compensation, and high SNR single-photon detection and screening, to extend the transmission distance limit of the repeater-less QKD system up to 830 km [19]. However, it should be noted that typical TF-QKD systems require photon level interference control between long-range independent lasers, which imposes harsh requirements on light source frequency locking and channel fluctuation compensation. The TF-QKD system was still under development in the laboratory, and no commercial products or implementation solutions were provided.

To further enhance the key rate and performance of the QKD system, different multiplexing techniques, such as time division multiplexing (TDM), wavelength division multiplexing (WDM), and space division multiplexing (SDM), could be considered to realize the parallel transmission of multiple QKD channels. Although time multiplexing based on optical path switching introduced a small amount of channel loss, the redundancy of the QKD system could guarantee the point-to-point key rate [40]. Multi-wavelength WDM or [41] SDM of multi-core or few-mode fibers [42] could provide solutions for QKD systems to transmit in parallel in the same fiber and enhance the final key rate.

Different from DV-QKD protocols that use weakly coherent pulsed quasi-single photons to encode quantum states, CV-QKD protocols typically use two-dimensional Gaussian modulation (GM) of quantum coherent states [43]. On one hand, CV-QKD systems can utilize conventional optical communication components, such as IQ modulators and coherent detectors, which are more advantageous in terms of miniaturization and cost reduction. On the other hand, CV-QKD can achieve a high security key rate of Mbps at tens of kilometers transmission distance. It is expected to become the mainstream technology for metro-area QKD solutions.

After 20 years of development, CV-QKD protocols mainly focused on GG02 [44], No-Switching [45], and discrete modulation [46], while the proof of protocol security has been completed [47]. System architecture evolved from transmitting local-oscillation, to local local-oscillation and discrete digital modulation systems. In 2022, the ISC team obtained a 21.53 Mbps secure key rate in a single-carrier four-state discrete digital modulation CV-QKD system experiment at a distance of 25 km [48], and a probabilistic shaping 256 QAM discrete modulation and digital coherent demodulation CV-QKD system to achieve a 9.193 Mbps secure key rate at a distance of 50 km [49]. Although the hardware of discrete digital modulation CV-QKD system was relatively simple, it relied on a high-performance DSP for coherent demodulation, signal compensation, and high-throughput post-processing. Until now it was still in laboratory development stage, and commercialized products have not been widely available.

Because of the advantages of high-dimensional quantum states (qudit) such as higher information capacity and noise resilience, high-dimensional QKD was another frontier research hotspot [50]. The qudit state preparation and QKD based on various degrees of freedom such as OAM, time, frequency, and time-bin have been demonstrated in various scenarios [51], such as fiber optic channels [52], including multimode and multicore fibers, etc., free-space channels [53], and underwater channels [54]. The high-dimensional encoding experiments in the MDI-QKD protocol were also verified [55]. It should be noted that the generation and control of qudit still had some limitations and problems in both theory and experiment, and the reliance on devices and channels such as integrated photonics and multicore/multimode fibers also posed challenges to the practical application of high-dimensional QKD.

### 2.2. Satellite-Based QKD Experiments

Satellite platforms for satellite-ground quantum communication and QKD have unique advantages in scientific research and applications [56]. On the one hand, as QKD terminals, satellites can significantly improve the transmission distance. The loss of the low earth orbit (LEO) satellite to the ground downlink channel is only about 40–50 dB, which is more than 10 orders of magnitude lower than that of fiber channel loss at the same distance [57]. Before the practical use of quantum storage and quantum relays, satellite platforms are the only solution to achieve thousands of kilometers of quantum communication. On the other hand, as QKD relay node, satellites can realize on-demand networking with ground stations worldwide, which has the advantages of mobility, coverage, and survivability, and thus can enhance the security of the QKD key relay function.

In recent years, a few countries and regions around the world have started to fund and implement satellite quantum communication and QKD research and application projects. Canada’s QEYSSat project, which has received tens of millions of dollars investment, collaborated with Honeywell on an earth-to-satellite quantum communication uplink containing a ground-based quantum source and miniaturized satellite receivers [58]. The CubeSat-based Quantum Communication Mission (CQuCoM), a joint effort by National University of Singapore and several other institutions, used the CubeSat platform deployed from the International Space Station for entangled photon transmission and QKD experiments to demonstrate a high-performance light source and pointing mechanism to establish satellite-to-ground entangled distribution, and tried to establish the basis for quantum constellation of LEO trusted relays [59]. The University of Grenoble, France, has proposed the Nanobob nano-satellite program to realize the uplink configuration for quantum communication from ground-to-satellite, while conducting research such as precise clock synchronization [60].

In 2016, USTC collaboration with other Chinese research institutions launched the first quantum science experiment satellite Micius and carried out a number of groundbreaking space quantum communication experiments in the following six years. The main technical achievements of Micius are shown in Table 2.

It should be noted that satellite-based quantum communications and QKD require solving a series of engineering challenges such as satellite acquisition, tracking and pointing (ATP), real-time channel compensation, and satellite-ground synchronization, as well as fulfilling requirements of weather conditions, all-day operation, and reliable maintenance. Due to limitation of daylight background noise and its own orbit altitude, the Micius satellite could only transmit quantum state signals with ground station between a short window (several minutes per day) on clear nights. In the future, by using a 1550 nm wavelength source combined with up-conversion detectors, it could be expected to improve detection efficiency and achieve day-time operation.

## 3. QSC Application Exploration in China

Over the past decade, with the increasing maturity of QKD technology, QKD trial network construction and QKD-based QSC application exploration have been widely conducted in many countries and regions around the world [21,71,72]. In China, steady progress has been made in QKD-based QSC application exploration, such as converging quantum encryption with multiple ICT protocols and systems, QKD network construction and demonstrational applications, and QSC applications based on QKD satellites.

### 3.1. Quantum Encryption and ICT Systems Integration

The key exchange mechanism (KEM), digital signature (DS) and authentication mechanism in public key cryptography, such as RSA and ECC, and symmetric encryption algorithm, such as AES, can guarantee the integrity, non-repudiation, and confidentiality of information [73]. To address the quantum computing security threats to existing public-key cryptography, quantum encryption based on QKD and/or quantum random number generators (QRNG), as well as novel key exchange mechanisms and digital signature algorithms in PQC, can be integrated with ICT systems and networks in different ways, as shown in Table 3.

Cryptographically secure pseudo-random number generators (CSPRNGs) are commonly used as random entropy sources for various algorithms. Using a quantum random number generator (QRNG) as an entropy source or fusing the output random numbers with a PRNG can enhance the randomness and performance [74].

If large-scale quantum computers are realized, current KEM and DS based on factoring, discrete logarithms, and elliptic curve cryptography will be at risk. In contrast, symmetric cryptographic, such as AES and hash functions, would not be as drastically impacted [75]. Using PQC to upgrade KEM and DS that face upcoming risk has become an important effort by ISO and industry. It should be noted that transition and upgrade of PQC requires the support of security-proof algorithm standards and reliable commercialized products, as well as considering algorithm performance, ease of implementation, compliance, etc. For the upgrade of a large number of ICT system devices, it will take a long time to complete [76].

QKD can provide a novel ITS solution for KEM in high-security requirement scenarios and where fiber resources are available, using the QKD system or network to provide symmetric key for encryption is a typical QSC use case. In practical application of this use case, the quantum key generated by the QKD system, or the relay key generated by the QKD network, is invoked by the encryptor on demand as real-time updatable key primitives in symmetric encryption to participate in the working key and session key generation process, thus enhancing the overall encryption security. It should be noted that QKD only solved the KEM problem; DS and authentication still needed the assistance of current encryption algorithms or PQC. Due to the secure key rate limitation, QKD keys are usually used as primitives in symmetric encryption algorithms, e.g., ASE, and further involved in session key generation [77]. Furthermore, in actual application, demand for fiber resources for the QKD system, the deployment, calibration and maintenance of hardware, and the sensitivity of the system to environmental impacts, such as fiber and equipment vibrations and ambient temperature fluctuations, are possible problems that lead to difficulty of commercializing and promoting QKD.

In order to implement the so-called ITS encryption, quite demanding requirements should to be met, for example, random entropy source based on QRNGs and KEM based on a practical security-verified QKD system or network, while key storage and forwarding based on trusted nodes should be avoided by using only pre-shared keys and universal hash checks for DS and authentication, and using only one-time-pad (OTP) for encryption processes. Use cases meeting all the above requirements are very limited and have more theoretical significance than practical value.

### 3.2. Application Schemes of Quantum Keys

**Figure 1:**
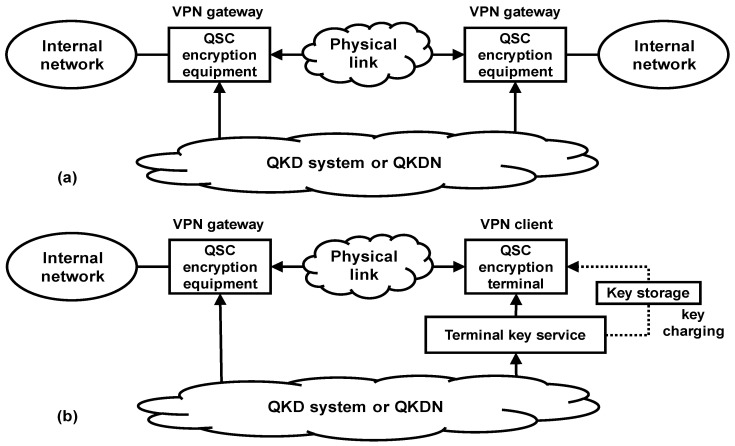
QKD key service schemes in QSC encryption. (**a**) Typical QSC between virtual private network gateways. (**b**) The offline quantum key services.

In QKD-based QSC, providing an end-to-end quantum key or relayed key for different types of encryptions is the basis for expanding use cases and commercial opportunities. Typical QSC between virtual private network (VPN) gateways, as shown in Figure 1a, could initiate key requests from the QKD system or network directly, and obtain quantum keys or relay keys on-line. At this time, the security of symmetrical keys is related to practical security of the QKD system and QKD network (QKDN), which requires standardization and verification to ensure. These use cases are the mainstream of QSC applications. Different types of quantum encryption VPNs, and routers, etc., have appeared and been deployed in multiple experimental and demonstration networks [78].

For more application scenarios which could not directly obtain quantum keys from QKD systems and QKDN, with assistance of key charging and storage schemes, one can realize offline quantum key services, as shown in Figure 1b. Terminal key service (TKS) was responsible for quantum key charging and storage functions, as well as synchronization and certification between the encryption equipment and terminals [79]. Based on the offline QKD key service, so-called quantum-encrypted mobile phones and customer premise equipment (CPE) have started tentative commercialization promotion in several network operators and infrastructure providers [80]. It should be pointed out that the security of final symmetry keys may be degraded and not meet the ITS requirement, due to the additional key storage and interaction function of TKS.

After obtaining quantum keys, how to effectively integrate them into encryption algorithms is also critical to support QSC applications. IPSec, MacSec, TLS, OTNSec, and other encryption protocols usually have self-negotiated key mechanisms based on public-key cryptography, which also contain functions such as security alliance establishment and identity authentication in order to ensure integrity and non-repudiation of information. Therefore, it was not feasible to use quantum keys to directly replace the self-negotiated key in the above protocols.

The QKD key could be used as a special pre-shared symmetrical key and mixed with a self-negotiated key in various encryption protocols to generate quantum-enhanced hybrid session keys. The combination of different keys, by XOR function or stirring function based on abstract operation, could provide better compatibility and reliability, especially when the QKD key was not available because of system or fiber channel failure. Key hybrid protocols could be easily implemented in software, while acquisition of quantum keys from QKD systems or QKDNs needs application interface and protocol support, which should be standardized to ensure internetworking.

### 3.3. QKD Network Construction and Application

Based on the quantum key generation function of the point-to-point QKD system, key storage and relay function of trusted nodes, and the key routing and networking function of the network controller, end-to-end quantum key service of the QKDN could be realized. Forming large-scale so-called “quantum key infrastructure” is the most ambitious target of the QKD industry. 

Since the first 125 km commercial fiber QKD emerged in 2004 [81], Chinese teams from both academia and industry have completed a number of QKD network constructions and demonstrational applications [82,83,84]. In 2016, the Beijing–Shanghai Backbone project built a quantum secure communication backbone for connecting Beijing and Shanghai, via Jinan, Hefei and other places, running in a total length of more than 2000 km, connecting metro-area networks of various cities and creating a large-scale quantum communication technology verification and application demonstration platform [65].

Based on the achievements of the Beijing–Shanghai Backbone, a larger-scale wide area QKD network was further under construction. The national QKD network project with a total length of over 10,000 km could connect major metropolitans such as Harbin, Wuhan, Chengdu, and Guangzhou, and also form a ring network in the eastern China areas, thus enhancing accessibility, service capability, and reliability of the entire QKD network.

In QKD metro-networks [85] such as Hefei and Jinan, dozens of user nodes, including government departments, financial agencies, and research institutes, are combined with trusted nodes through star-type or ring-type networking to form QKD services and provided quantum-encrypted real-time voice communication, file transfer, etc.

In addition to China, a number of QKD network construction projects and demonstration applications have been carried out in Europe, another important region for conducting QKD-based exploration of quantum secure communication applications. Since 2008, several QKD networks have been experimentally validated in Austria, Switzerland, Spain, and France [23]. In 2019, the Open European Quantum Key Distribution Testbed project supported more than twenty EU projects and teams to conduct experiments on QKD networks and cryptographic applications. Initial construction of inter-European quantum networks for deployment and applications have begun [86].

During QKD network construction and deployment, it is very important to share the existing fiber communication network infrastructure by performing wavelength division multiplexing between QKD and optical communication systems, e.g., OTN. Quantum signals are very weak and susceptible to classical signal impairments, such as spontaneous Raman scattering. Careful selection of the optimal wavelength of the quantum signal, reduction of classical optical signal launch power, and additional specially designed time and frequency domain filters are needed to achieve co-propagation between QKD and classical optical signals in areas of tens of square kms. [87]. However, since quantum signals could not pass through optical amplifiers such as EDFA, the co-propagation of QKD and OTN systems is limited to point-to-point links, and long-distance and multi-span integration is still very challenging.

### 3.4. QSC Application Based on QKD Satellites

In addition to the above-mentioned scientific experiments, the Micius satellite combined with ground fiber QKD network has verified the feasibility of the space- and ground-integrated quantum communication network [65]. By improving the operating frequency, telescope size, and coupling efficiency of the ground station, and using the optimized unbalanced basis selection protocol, the QKD key rate of a single orbit (about 6 min) under ideal weather conditions was up to 47.8 kbps, and the maximum satellite-relayed QKD key was about 36 Mbit per week [65]. 

To take advantage of the mobility and flexibility of satellite-based QKD, a portable ground station is essential supporting equipment. Portable ground stations weighing less than 100 kg, requiring less than 1 m^3^ of space, and taking no more than 12 h to install have been successfully developed and could be deployed on the rooftops of urban buildings to complete space-to-ground QKD experiments with Micius [88]. Satellite-based QKD is one of the most important use cases to fully utilize the advantages of QKD, which could provide quantum key services for remote locations or moving objects that do not have fiber accessibility.

It should be noted that satellite-based QKD applications still face many technical and engineering challenges. Micius is a LEO satellite having a limited transmission time window and ground coverage in a single orbit, and it could only work at night due to the limitation of light source working wavelengths and solar background noise. As a result, Micius was mainly used to verify the feasibility of space-to-ground QKD while its practical capability could not to be expected too much.

In recent years, there has been some progress to enhance satellite-based quantum communication capabilities, such as realizing daytime free-space QKD to overcome the effect of sunlight scattering background noise. By using a 1550 nm wavelength light source and detector, the intensity and scattering of daylight could be effectively avoided, and by combining a narrow bandwidth grating filter and an ultra-low noise up-conversion single photon detector, the background noise could be further reduced and 20 bps key rate QKD was achieved [89].

To realize a global space-to-ground quantum communication and QKD network, it was still necessary to further increase the number of satellites and heighten the orbit altitude to form a quantum constellation combining LEO and geosynchronous orbit (GEO) satellites. In 2022, a new QKD nano-satellite “Jinan-1” was successfully launched, weighing only 1/6 of the Micius., with about six times higher light source frequency, and capability to accomplish post-processing and key generation in real time [90]. In the future, it can be expected that the nano-satellites and portable ground stations will carry out more interesting QKD experiments and demonstrational QSC applications.

## 4. QSC Standardization in CCSA

With the advancement of QKD technology, development of commercialized products by multiple vendors, and exploration of QKD network construction and QSC application, the QKD-based QSC industry was initially formed in China, which includes research institutions, system vendors, network and service providers, and encryption service users, etc. In the QSC industry, standardization is an important segment to promote QKD network construction and deployment as well as QSC large-scale application, and has become a common concern to the management agencies, academia, and industry [91].

CCSA is the platform responsible for the research of China’s ICT standards system and specification development [92]. In 2016, CCSA established the Special Task Group on Quantum Communication and Information Technology (ST7). It is responsible for the standardization of quantum communication technologies and quantum communication networks, quantum computing technologies related to quantum communication, and general quantum information components. CCSA-ST7 brought together more than 60 stakeholders in the Chinese QSC market. Up to December 2022, it has established and carried out the development of 25 national and industrial standards related to quantum secure communications, as shown in Table 4.

### 4.1. QKD System and Component Standards

The specifications on minimum functional and performance requirements for QKD systems and components can provide vendors guidance on product design and development. At the same time, they can also provide users the necessary support to procure products, build networks, and encrypt applications.

QKD systems are the core of QKD network and QSC applications, and their functional and performance technical requirements and test methods are a priority for standardization. DV-QKD systems based on the decoy state BB84 protocol are the mainstream products in the Chinese QSC market. YDT 3834.1 and YDT 3835.1 standards specify system application code, model and reference points, system performance parameters, technical requirements of QKD transmitter and receiver, reliability and environmental adaptability, as well as the corresponding test methods. In addition, CV-QKD systems based on Gaussian modulated coherent state protocols, such as GG02 and No-switching, have been preliminarily commercialized, and similar specifications are also under development.

Developing technical specifications for key components in QKD systems could help to enhance the engineering and integration levels while promoting cooperation in the industry chain between component suppliers and system vendors. YDT 3907 series standards, which specified key components such as light sources, single photon detectors, QRNGs, decoy state modulators, quantum state modulators, and demodulators used in DV-QKD systems, cover optoelectronic characteristics, operating conditions, external dimensions, and corresponding test methods.

QRNGs can be used as QKD system components or as standalone products to provide better security and performance of random entropy sources for cryptographic applications, such as databases and cloud computing [93]. QRNGs have emerged in the Chinese market with a variety of products based on different technologies, such as phase rise and fall, vacuum noise, amplified spontaneous radiation noise, etc. QRNG product standards will regulate the system technical scheme, functional model, technical requirements, performance parameters, interfaces, reliability requirements, and related test methods.

### 4.2. QKD Network Standards

The QKD network is a supporting platform to realize end-to-end quantum keys service, and also a crucial step must be taken to break through the limitations of the QKD point-to-point application mode [94]. With the progress of QKDN construction and demonstration applications, network-related standardization has been carried out in international standard development organizations, such as ITU-T and ETSI [95]. In CCSA-ST7, specifications for QKD network architecture, protocol, interfaces, management, and key management are also being studied and developed.

The QSC network architecture standard refers to the ITU-T Y.3800 series of recommendations, specified functional architecture, network elements model and function, reference points, and network configuration procedure of QKD-based QSC networks. It also provides a framework overview for QKDN and QSC networks, and lays the foundation for subsequent network-related standards.

Trusted nodes are unavoidable functional modules of the QKD network at present, and also a focus of concern about practical security of QKD networks. Their specification and verification are an important basis to ensure performance and security of QKD networks. The technical requirements standard for trusted nodes in QKDNs could provide specifications for system composition, functional processes, interface management, and security requirements.

As the interaction interface for the QKD network to provide keys to the user network, the application interface of the QKDN is critical to realize quantum key service and network interoperability, and also the priority of QKD network standardization. The QKDN key service interface standard not only clarifies the application interface, function definition, and business interaction flow between key managers and application terminals but also provides a useful reference for QSC users to obtain quantum keys.

### 4.3. QSC Application Standards

Exploring and expanding the applicable scope and use cases of QSC based on QKD are necessary to promote the development of the industry. National standards named quantum secure communication use cases and requirements, described application of QKD in various layers and protocols in ICT systems, such as link layers, network layers, transport layers, and application layers, and clarified application schemes of QSC in multiple scenarios, such as data center interconnections, enterprise private networks, infrastructure information systems, telecommunications backbone networks and access networks, and satellite networks, etc., while they specified fundamental requirements such as security, scalability, robustness, and interoperability.

Quantum keys provided by QKD systems and networks need to be integrated with different protocols such as IPSec, TLS, VoIP, and OTNSec in various types of cryptographic application equipment, such as VPN gateways or routers, to complete QSC service. With updated quantum key acquisition and mixing protocols briefly described in Section 3.2., quantum encryption functions could be implemented while keeping the original encryption functions as backup. In order to guide equipment development and testing, specification of QSC encryptors based on IPSec protocols define quantum key acquisition and mixing protocols, encryption algorithms, function and performance requirements, and related test methods for gateways and terminals. Similar specifications of quantum encryptors based on TLS, VoIP and OTNSec protocols are also under development.

## 5. QSC Testing and Verification Practice

In the QSC industry, testing and verification are another important segment to promote QKD-based QSC network deployment and application. Based on the function and performance requirements in technical specifications and corresponding testing and verification methods, QKD-based QSC systems and networks could be tested and evaluated to provide users with performance, quality, and reliability assurance, and support their commercial product procurement and application solution deployment.

It should be noted that testing and evaluation of practical security of QKD systems and networks are also a very critical aspect of verification practice [96]; however, the study on QKD security-related standards is still in process, and reference bases for standardized verification are not yet completely adequate, which require more collaborative efforts in the industry. At present, market-oriented testing and verification mainly focus on function, performance, and reliability of QKD-based QSC systems and networks.

### 5.1. QKD System Test Evaluation

Based on the standards of technical requirements and test methods for the decoy state BB84 protocol DV-QKD system described in Section 4.1, the test evaluation of QKD systems has been carried out in the Chinese market; its test items are shown in Table 5. Recently, typical commercialized products of mainstream system vendors in the Chinese market have been tested and certified.

In QKD system testing, security key rate is the top system parameter of concern. Since key rate is related to transmission distance and channel loss, specifying application code in terms of typical channel loss such as 10 dB and 20 dB, is an effective way to measure the performance of QKD systems. By standardizing methods and formulas used in the post-processing process, including basis comparison, QBER calculation, error correction and privacy amplification, key rate comparison for QKD systems can be conducted with different implementations. The quantum key output from the QKD system should be verified by randomness testing in accordance with standards to guarantee security of symmetric keys.

For QKD transmitters and receivers, optical characteristics of quantum channel, synchronization channel, and distillation channel are closely related to the deployment and implementation of QKD networks, which need to be tested at the system level to provide accurate reference for applications. Meanwhile, verification on the accuracy of decoy state and quantum state modulation, such as the intensity fluctuation of signal and decoy states, quadrature and conjugate error of quantum state modulation, and difference of pulse time and frequency domain characteristics, could partly provide supporting evidence for practical system security. In addition, the single-photon detector (SPD) is the main limiting factor of QKD system performance and also an important parameter in secure key rate calculation. It is necessary to test and verify the performance parameters of the SPD, including detection efficiency, post-pulse probability, and dark count rate, etc.

The quantum-state optical signal of QKD systems is extremely weak, usually below −70 dBm. Commercial QKD systems need to be deployed in the same environment with other optical communication systems, leading to high demands on reliability and environmental adaptability, which are usually ignored in lab experimental or field trials. Verification of commercial QKD system reliability such as long-term stability, system redundancy protection, fault recovery capability, and robustness under different temperature and humidity conditions is the basis for ensuring continuity of service.

Through the development of standards and test verification, one can provide useful guidance and promotion for QKD systems to evolve from research-oriented prototypes to application-oriented mature commercialized products. It is reasonable to believe that the engineering and practical level of QKD systems will be further enhanced, with continuous progress of standardization and test verification.

### 5.2. QKD System Test Evaluation

Connecting multiple QKD systems to build QKD networks is important to extend and enhance the capability and scope of key services. The quantum keys generated by point-to-point QKD links are synchronized by key-ID, authenticated, and stored by key managers (KMs) deployed in trusted nodes. Then the quantum keys are further relayed hop-by-hop through the classical communication channel between KMs to provide end-to-end symmetric keys, typically using OTP encryption during the relay to maintain the key’s ITS level. The above networking functions at different layers of the QKDN are accomplished with the help of network management and the controller; the QKD network framework and functional architecture should conform to the requirements of ITU-T Y.3800 series Recommendations [97].

The relay key provisioning capability of an end-to-end link is limited by the minimum value of key rate of all point-to-point QKD links within it; therefore, it is necessary to test and verify the actual key rate of all QKD links in the network. The channel loss of legacy fiber network may differ from nominal value due to various factors, such as station distance and fiber cable status, which could affect QKD key rate. It is a common solution to deploy multiple pairs of QKD systems simultaneously in a high channel loss fiber link to ensure the key rate meets the design requirements. It should be noted that this stacked QKD system deployment is not typical redundant protection used in optical communication networks, such as 1 + 1 or 1:1 protection, because all systems are in working condition together to guarantee the key rate of point-to-point links.

The protection and recovery of QKD networks are mainly realized in the key management layer, which usually requires multiple key relay paths or the formation of a ring-type network to provide backup routing support. Protection of QKD networks requires classical communication systems such as OTNs and routers to support network management and QKD distillation, which should have capabilities for their own protection and recovery. Furthermore, it also requires capabilities based on network controllers and management to realize re-routing function of key relaying. Since the KM has a caching function, upper-layer key applications are usually unaffected to the protection recovery process of key-relay rerouting. For large and complex topology QKD networks, verification of protection and recovery capabilities at both levels of classical communication and key relaying is important to guarantee QoS of key provisioning.

Necessary service support systems and networks in QKD networks, such as OTNs, IP networks, time synchronization, and network management, etc., are the basis for guaranteeing overall service quality and also need attention in network-level test verification. The planning and configuration of OTN and IP networks, multi-service support capability, and long-term stability of service are major concerns. NTP-based network time synchronization provides millisecond-level timing accuracy and supports life cycle management of keys, network performance, fault monitoring, service billing, and other management functions. Time deviation and redundancy protection for NTP time servers and clients need to be tested and verified to ensure reliability of overall time service capability of QKD networks. Other network-level tests include verification of functions such as network management systems, business support, and application service platforms.

## 6. Discussion and Outlook

During the past two decades, QKD technology research has remained active, performance such as transmission distance and key rate of fiber-based QKD systems have been significantly improved, and satellite-based QKD has completed feasibility verification. Commercialized QKD-based QSC systems have been carried out by many vendors, network construction has been carried out in many regions around the world with cryptographic application exploration in government, finance, infrastructure, etc. Standardization of QKD and QSC devices, systems, networks, services, and security have been carried out in several international and regional SDOs. Based on the relevant standards, test verification and certification of QKD systems and networks are also ongoing. In the foreseeable future, the momentum of QKD-based QSC technology development and application exploration will continue.

However, from the industry perspective, it is unfortunate that the application and commercialization of QKD-based QSC has not experienced the so-called “exponential growth” over the past decade, and the financial data and capital market performance of related companies were fairly lackluster. Any level-headed stakeholder will acknowledge that the application and commercialization of QKD-based QSC still face obstacles and challenges, some of which are listed as below:

1. Technology: In terms of protocol mechanism, quantum state signals in QKD systems sacrifice the robustness of transmission in exchange for the security of key generation, which is a crucial barrier to further improvement of transmission capability and key rate, and also fundamentally limits their adaptability and reliability in practical environments outside the laboratory. Although the performance of QKD systems based on new protocols such as TF and MDI have been improved, and satellite-based QKD has been proven to be technically feasible, there is still a long way to go for the commercial products based on these protocols and platforms to be applied on a large scale.
2. Application: For QKD-based QSC application, firstly, almost-dedicated fiber resources to support deployment of hardware system are needed; secondly, it may involve change or integration of the user’s network architecture, equipment, and service routing; thirdly, highly specialized configuration and maintenance management of QKD systems are also needed; finally, QKD systems are still relatively expensive. All the above issues will become capital expenditure and operating expenses that users have to be concerned about in adopting this technology. QKD technology and system development based on integrated photonics can improve the integration and robustness of the system, while reducing system cost to enhance scalability, which will be very beneficial for expanding QKD applications.
3. Standards and Certification: Although important progress has been made in QKD-related standards, much work remains to be done. One of the most significant is credible specification and test verification for practical security proof of QKD systems, which are essential to fully guarantee the ITS advantage and convince customers with high security requirements. In addition, specifying interfaces and protocols for KM layers in QKD networks to facilitate cross-domain interoperability may be another priority for future standardization.

In summary, QKD has unique advantages of secure symmetric key distribution between remote locations, which has a wide range of applications in cryptography, such as encryption and authentication, as well as guaranteeing long-term security in the era of quantum computation. QKD has been supported by both academia and industry, and technical research has made steady progress from the theoretical protocols of 40 years ago to nowadays with thousand km transmission and key sharing. With the utility of new protocol systems, the miniaturization of PIC-based systems, and the maturity of commercialized products, QKD-based QSC deployment and application will become more widespread in high-security-requirement network communication scenarios. The development of standardization and test verification will also provide useful guidance and support for its industrialization. In the quantum era, it is reasonable to be cautiously optimistic about the future development and application of QKD-based QSC.

## Figures and Tables

**Table 1:** Typical QKD experiments and their performance.

Protocol	Channel	Distance or Loss	Key Rate (bps)	Year	Reference
Modified BB84	Lab fiber	421 km	6.5	2018	[29]
Twin-field	Lab fiber	90.8 dB	0.045	2019	[31]
Twin-field	Lab fiber	502 km	0.118	2020	[35]
Twin-field	Lab fiber	509 km	0.269	2020	[36]
Twin-field	Lab fiber	605 km	0.97	2021	[37]
Twin-field	Field Trial	511 km	3.45	2021	[38]
Twin-field	Lab fiber	658 km	0.092	2022	[39]
Twin-field	Lab fiber	830 km	0.014	2022	[19]

**Table 2:** Micius quantum science satellite experiments.

Experiment	Achievement	Year	Reference
Quantum Key Distribution	1200 km satellite-to-ground QKD (1.1 kbps key rate)	2017	[61]
	1000 km satellite-to-ground entanglement-based QKD (3.5 bps key rate)	2017	[62]
	7600 km apart ground stations satellite relay QKD and encryption demonstration (key volume 100 KB)	2018	[63]
	1120 km apart ground stations entanglement-based QKD (0.12 kbps key rate)	2020	[64]
	Increasing key rate 40 times in satellite-ground QKD (47.8 kbps key rate)	2021	[65]
Quantum Teleportation	1200 km apart ground stations entanglement distribution (0.869 fidelity)	2017	[66]
	1400 km ground-to-satellite quantum teleportation (0.80 fidelity)	2017	[67]
	1200 km apart ground stations quantum state transfer (0.82 fidelity)	2022	[68]
Quantum Physics	Experimental of gravitationally induced quantum decoherence model	2019	[69]
	Satellite-to-ground quantum-secure time transfer (9 kHz time data rate, and 30 ps transfer precision)	2020	[70]

**Table 3:** Quantum encryption and ICT systems integration.

Solution	Entropy Source	Key Establishment	Digital Signature	Encryption
Current	CSPRNG *	ECDH (SM2 **)	RSA (SM2)	AES (SM4 **)
PQC	CSPRNG	PQC KEM ***	PQC DS ****	AES (SM4)
QRNG + PQC	QRNG	PQC KEM	PQC DS	AES (SM4)
QKD-based QSC	CSPRNG or QRNG	QKD	RSA (SM2) or Pre-shared Key	AES (SM4)
QRNG + QKD + PQC	QRNG	QKD	PQC DS	AES (SM4)
ITS Encryption	QRNG	QKD	Pre-shared Key	OTP

* CSPRNG: Cryptographically secure pseudo-random number generator. ** SM2/SM4: Commercial cryptographic algorithms standard in Chinese market. *** PQC KEM: CRYSTALS-KYBER, BIKE, Classic McEliece, HQC, SIKE [14]. **** PQC DS: CRYSTALS-Dilithium, FALCON, SPHINCS+ [14].

**Table 4:** Quantum secure communication standardization progress in CCSA-ST7.

Type	Subject	Status
National	Quantum communication terms and definitions	Ongoing
National	Quantum secure communication use cases and requirements	Ongoing
Industrial	Quantum key distribution (QKD) system technical requirements Part 1: BB84 protocol-based QKD system	Released 2021
Industrial	Quantum key distribution (QKD) system test methods Part 1: BB84 protocol-based QKD system	Released 2021
Industrial	Quantum key distribution (QKD) system application interface	Ongoing
Industrial	Components for BB84 protocol quantum key distribution (QKD) Part 1: Light source	Released 2022
Industrial	Components for BB84 protocol quantum key distribution (QKD) Part 2: Single photon detector	Released2022
Industrial	Components for BB84 protocol quantum key distribution (QKD) Part 3: Quantum Random Number Generator (QRNG)	Released 2021
Industrial	Quantum secure communication network architecture	Released 2022
Industrial	Quantum key distribution and optical communication co-propagation technology requirements	Ongoing
Industrial	Quantum key distribution (QKD) network interface requirements between key management and QKD	Ongoing
Industrial	Quantum key distribution (QKD) equipment security requirements Part 1: QKD based on decoy state BB84 protocol	Ongoing
Industrial	Technical specifications for quantum secure communication application equipment based on IPSec Protocol	Released 2022
Industrial	Quantum key distribution network management system technical requirements	Released 2022
Industrial	Quantum key distribution (QKD) system technical requirements Part 2: Gaussian modulated coherent state protocol-based QKD	Ongoing
Industrial	Quantum key distribution (QKD) system test methods Part 2: Gaussian modulated coherent state protocol-based QKD	Ongoing
Industrial	Technical requirements for trusted nodes of quantum secure communication networks	Ongoing
Industrial	Quantum key distribution network security technology requirements	Ongoing
Industrial	Components for BB84 protocol quantum key distribution (QKD) Part 4: Decoy state modulation module	Ongoing
Industrial	Components for BB84 protocol quantum key distribution (QKD) Part 5: Quantum state modulation module	Ongoing
Industrial	Components for BB84 protocol quantum key distribution (QKD) Part 6: Quantum state de-modulation module	Ongoing
Industrial	Quantum random number generator technical specifications	Ongoing
Industrial	Transport layer cryptography protocol-based quantum secure communication application equipment technical specifications	Ongoing
Industrial	Technical specification for quantum secure communication application equipment for VoIP services	Ongoing
Industrial	Quantum key distribution (QKD) network technical requirements for key management	Ongoing

**Table 5:** QKD system testing and evaluation according to YDT 3834.1/3835.1.

QKD Test Objects	QKD Test Items
System performance	Average secure key rate of QKD
	System channel-loss margin
	QKD output key consistency
	QKD output key randomness
QKD transmitter	Optical source time-domain characteristics
	Optical source frequency-domain characteristics
	Random number generator characteristics
	Decoy state modulation time-domain characteristics
	Decoy state modulation probability distribution
	Quantum state modulation time-domain characteristics
	Quantum state modulation frequency-domain characteristics
	Quantum state modulation demodulation accuracy
	Average photon number of quantum state signal
	Injection optical isolation
QKD receiver	Injected light leakage threshold
	SPD time-domain response characteristics
	SPD dark count probability
	SPD dead time
	SPD detection efficiency
	SPD post-pulse probability
Synchronization channel	Optical signal time-domain characteristics
	Optical signal frequency-domain characteristics
	Optical signal receipt sensitivity
Distillation channel	Optical signal time-domain characteristics
	Optical signal frequency-domain characteristics
Other system features	System long-term stability
	System redundancy protection
	System start-up time
	System recovery time
	System environmental reliability
	Power supply tolerance
Network management	System management features
	Network management features

## Data Availability

No new data were created.
